# Meteorological Register for October, 1816

**Published:** 1816-12

**Authors:** John Bailey

**Affiliations:** Surgeon, &c.


### Meteorological Register for October, 1816.

*Kept at Harwich, in Essex.*

By JOHN BAILEY, Surgeon, &c.

| Moon's Age. | Atmospherical Pressure. Morn. | Ev. | Temp. | Wind. | Remarks. | General Statement of Diseases. |
|---|---|---|---|---|---|---|
| 1 | 29 5½ | 29 4 | 60 | W | Rain. Gale of w. | |
| 2 | 6 | 4 | 60 | W | ditto | |
| 3 | 3 | 3 | 60 | N | Fine | Intermittentes |
| 4 | 8 | 8 | 62 | SE | Rain | |
| 5 | 8 | 8 | 62 | W | Fine | Scarlatina |
| ○ 6 | | | 67 | W | ditto | |
| 7 | | | 61 | E | Rain. Tempest | Pneumonia |
| 8 | ½ | 9½ | 64 | E | Fine and clear | |
| 9 | 30 | 30 | 60 | W | | |
| 10 | | | 61 | SW | | |
| 11 | 29 9 | 30 | 59 | W | Foggy | |
| 12 | 30 ½ | ½ | 58 | N | | |
| 13 | | | 58 | SW | | |
| ☾ 14 | | | 56 | SW | | |
| 15 | 1 | 1 | 60 | SW | | |
| 16 | ½ | 29 8 | 58 | S | | |
| 17 | 29 7½ | 7½ | 58 | W | Rain | |
| 18 | 7 | 7 | 49 | W | ditto | |
| 19 | 7 | 7 | 46 | W | | |
| ● 20 | | | 41 | NW | | |
| 21 | 8 | 8 | 48 | N | Rain. Gale of w. | |
| 22 | 30 | 30 | 50 | NW | | |
| 23 | 1 | 1 | 54 | SW | | |
| 24 | 29 8 | 29 6 | 50 | SW | ditto | |
| 25 | 5 | 5 | 50 | S | Rain | |
| 26 | 6 | 6 | 52 | SW | | |
| ☽ 27 | 6½ | 6½ | 55 | S | Rain | |
| 28 | 6 | 6 | 56 | E | | |
| 29 | 6 | 4 | 56 | SE | | |
| 30 | 2½ | 2 | 52 | E | Rain | |
| 31 | 2 | 2 | 50 | | ditto | |

During the greater part of this month, pneumonia has been of frequent occurrence; and in one instance, in despite of the most active means, fatal. Scarlatina still hangs about the neighbourhood. Intermittents are becoming prevalent.

---

END OF VOL. II.

